# What is Elective Oncologic Surgery in the Time of COVID-19? A Literature Review of the Impact of Surgical Delays on Outcomes in Patients with Cancer

**DOI:** 10.31487/j.COR.2020.06.05

**Published:** 2020-06-26

**Authors:** Denise Garcia, Julie B. Siegel, David A. Mahvi, Biqi Zhang, David M. Mahvi, E. Ramsay Camp, Whitney Graybill, Stephen J. Savage, Antonio Giordano, Sara Giordano, Denise Carneiro-Pla, Mahsa Javid, Aaron P. Lesher, Andrea Abbott, Nancy Klauber DeMore

**Affiliations:** 1 Department of Surgery, Medical University of South Carolina, Charleston, South Carolina, USA; 2 Department of Surgery, Brigham and Women’s Hospital, Boston, Boston, Massachusetts, USA; 3 Department of Obstetrics and Gynecology, Medical University of South Carolina, Charleston, South Carolina, USA; 4 Department of Urology, Medical University of South Carolina, Charleston, South Carolina, USA; 5 Department of Medicine, Medical University of South Carolina, Charleston, South Carolina, USA

**Keywords:** Time to surgery, COVID-19, outcomes, surgical delays, surgical oncology

## Abstract

**Background:**

The impact of the COVID-19 pandemic has spread beyond those infected with SARS-CoV-2. Its widespread consequences have affected cancer patients whose surgeries may be delayed in order to minimize exposure and conserve resources.

**Methods:**

Experts in each surgical oncology subspecialty were selected to perform a review of the relevant literature. Articles were obtained through PubMed searches in each cancer subtype using the following terms: delay to surgery, time to surgery, outcomes, and survival.

**Results:**

Delays in surgery > 4 weeks in breast cancer, ductal carcinoma in situ, T1 pancreatic cancer, ovarian cancer, and pediatric osteosarcoma, negatively impacted survival. Studies on hepatocellular cancer, colon cancer, and melanoma (Stage I) demonstrated reduced survival with delays > 3 months.

**Conclusion:**

Studies have shown that short-term surgical delays can result in negative impacts on patient outcomes in multiple cancer types as well as *in situ carcinoma*. Conversely, other cancers such as gastric cancer, advanced melanoma and pancreatic cancer, well-differentiated thyroid cancer, and several genitourinary cancers demonstrated no significant outcome differences with surgical delays.

## Introduction

The outbreak of the novel beta coronavirus known as acute respiratory syndrome coronavirus 2 (SARS-CoV-2) has dramatically changed the world. The first documented cases of COVID-19 occurred in December of 2019. On March 11, 2020, COVID-19 was declared a pandemic, and it has claimed over 299,596 deaths worldwide as of May 14, 2020 [1, 2]. This pandemic not only impacts those infected but also has consequences for all patients within the healthcare system. Among those impacted are cancer patients whose surgeries may be delayed and rescheduled to minimize their own exposure as well as to conserve hospital resources in efforts to build surge capacity.

These are critical concerns with ongoing decisions being made amidst this pandemic to delay elective surgeries, and it begs the question: what should we consider elective oncologic surgery? Further complicating the concern for worsened outcomes from delays in cancer treatment, is the risk of the COVID-19 infection itself. The timing of oncologic surgery must be carefully balanced with the increased susceptibility to infection and worsened outcomes in the cancer population infected with COVID-19 [3].

As community care centers and major academic institutions begin cancelling elective surgeries, surgical oncologists are finding themselves facing unprecedented decisions in patient triage. The decision to delay a cancer operation is not straightforward. It is a multifactorial decision based on disease-, patient-, and system-specific variables. In addition to patient concerns, there is a valid concern of placing the healthcare personnel at risk while operating on non-emergent cases during this pandemic.

Several national surgical societies have created guidelines for triage and surgical management of cancer patients in the era of COVID-19. The Society of Surgical Oncology has Disease Site Specific Management Resources COVID-19 to help guide management during these unprecedented times [4]. Other societies that have come out with guidelines for cancer patient surgical management include the American College of Surgeons, the American Society of Breast Surgeons, the American Association of Endocrine Surgeons, and the International Journal of Gynecologic Cancer [5–8]. It is also unknown how to prioritize patients for surgery when normal operations resume, and it is important to note that these organizations underscore the importance of factoring in patient and institutional preferences as well as community resources in these complex decisions. Knowledge of the impact that surgical delays may have on outcomes can factor into the prioritization of patients for surgery. For this purpose, we have performed a literature review that analyzes time to surgery with respect to cancer patient outcomes. This review highlights cancers that could be negatively impacted by surgical delays from this pandemic, as well as those that have not been observed to have worsened outcomes with increased time to surgery.

## Methods

The purpose of this comprehensive literature review was to help drive evidence-based clinical decisions on delays of oncologic surgery amidst the COVID-19 pandemic. Subspecialty experts in each surgical oncology subspecialty (Breast, Endocrine, Gastrointestinal, Hepatobiliary, Melanoma, Gyn/Onc, Pediatric, Sarcoma, Urology) were selected to perform a review of the literature in their area of expertise. Articles were obtained through PubMed searches in each cancer subtype using the following terms: delay to surgery, time to surgery, outcomes, and survival. Selected studies were required to specify a delay in surgery, indicate a time interval, and assess patient outcomes. If multiple studies were available within a cancer subtype, the more recent studies were selected based on treatment relevance, with a focus on systematic reviews and larger sample sizes. Additional studies were accessed as deemed pertinent by individual searches and validated by subspecialty experts. The review was then organized by cancer type. Each section was edited and reviewed by the respective subspecialty content expert.

## Results

### I Breast Cancer

Breast cancer is the most frequently diagnosed cancer and the second leading cause of cancer death in women [9]. Retrospective studies addressing diagnostic or treatment delays in breast cancer and their effect on outcomes are reviewed for patients with invasive carcinoma requiring upfront surgery, ductal carcinoma in situ (DCIS), and patients having surgery after completion of neoadjuvant chemotherapy.

#### i Invasive Breast Cancer, Excluding Neoadjuvant Therapy

Bleicher *et al.* used the Surveillance, Epidemiology, and End Results Program (SEER) and the National Cancer Database (NCDB) to evaluate the association of time to surgery (TTS) with overall survival (OS) at 30-day intervals [10]. The SEER analysis included 94,544 patients with Stage I-III breast cancer from 1992–2009, and the NCDB reviewed 115,790 patients with Stage I-III breast cancer. The increase in mortality in all Stages for all patients and from all causes was 9% (hazard ratio (HR), 1.09; 95% confidence interval (CI), 1.06–1.13; p < 0.001) for each preoperative time interval category increase for the SEER database and 10.0% (HR, 1.10; 95% CI, 1.07–1.13; p < 0.001) for the NCDB.

Two smaller studies have shown conflicting data. Mansfield *et al.* used the Ohio State Cancer Database to evaluate TTS in three groups up to 60 days and showed worse DFS in the early group in subset analyses by Stage [11]. The authors postulated that patients in the early surgery group might have had worse DFS due to biological risk factors known to the surgeons, which influenced their surgical timing and led to a selection bias. The authors concluded that surgery up to 60 days does not affect DFS. Another small study by Wagner *et al.* evaluated the relationship of TTS with tumor growth and nodal progression in 818 clinically node-negative patients and found no association in multivariate analysis [12].

Despite two smaller studies showing no impact, the study by Bleicher *et al.* is the most comprehensive study on the subject performed [10]. This study includes data from two of the largest cancer registry databases in the United States, and analysis of both demonstrate that increasing TTS starting at four weeks is associated with a reduction in OS.

#### ii Ductal Carcinoma in situ

Mansfield *et al.* reviewed The Ohio State Registry of 243 patients with DCIS and divided TTS into groups: <21 days, 22–42 days, and 43–63 days [11]. They found no difference in DFS between groups. A larger study performed by Ward *et al.,* evaluated the association delay had on progression to invasive disease. The study included 140,615 patients with DCIS from the NCDB from 2004–2014 [13]. OS and invasion were assessed by TTS for time intervals: ≤ 30, 31–60, 61–90, 91–120, 121–365 days. Pathologic analysis was performed to distinguish DCIS versus invasive breast cancer on final pathology. Among all clinical DCIS patients, 16,668 (11.9%) had invasive ductal carcinoma. Increasing delay to surgery was an independent predictor of invasion (odds ratio (OR), 1.13; 95% CI 1.11–1.15; P < 0.001). With each delay interval increase, added relative risk of death was 7.4% (HR 1.07; 95% CI 1.05–1.10; P < 0.001) [13].

There are currently three prospective randomized controlled active surveillance trials ongoing that are evaluating experimental arms to standard treatment for DCIS. The Low Risk DCIS (LORD) trial in Europe is testing active surveillance alone versus standard treatment; the Low Risk DCIS (LORIS) trial in Europe and the Comparison of Operative to Monitoring and Endocrine Therapy for low-risk DCIS (COMET) trial in the United States allow for endocrine therapy in the active surveillance cohort [14]. Exclusion criteria for these studies include mammographic or palpable mass, due to a known increase risk of invasion. In triaging patients for surgery during the COVID-19 pandemic, consideration of treating patients as if they were on the neoadjuvant endocrine arm of the COMET trial is being recommended by the Society of Surgical Oncology. When considering patients with DCIS for surgery versus neoadjuvant endocrine treatment, one must be thoughtful of the fact that 17–20% of patients with a core biopsy of DCIS are upstaged to invasive breast cancer on final pathology review after excision [14, 15]. Using high-risk criteria of breast mass on imaging or exam, ER-negative status, or grade III DCIS should be considered for surgery and weighted against the shortage of resources.

#### iii Surgery Following Neoadjuvant Chemotherapy

Sanford RA *et al.* reviewed the MD Anderson breast cancer database for Stage II and III patients undergoing neoadjuvant chemotherapy between 1995–2007 [16]. Time to surgery intervals were divided as follows: <4 weeks, 4–6 weeks, >6 weeks, for a total of 1101 patients. The 5-year OS estimates were 79%, 87%, and 81% in patients who underwent surgery at ≤4, 4–6, and >6 weeks after neoadjuvant chemotherapy respectively (p = 0.03). A sensitivity analysis comparing ≤8 weeks to a small group of 8–24 weeks (6.4%) presented worse outcomes when surgery was performed after a time interval greater than 8 weeks.

Suleman *et al.* evaluated patients with Stage II and III breast cancer undergoing neoadjuvant chemotherapy between 2004–2014 [17]. TTS was divided into groups: < 4 weeks, within 4–7 weeks, and ≥8 weeks after completion of chemotherapy. The 5-year OS rate was not statistically significant between groups. However, only 12.9% of the patients who received surgery after ≥8 weeks had pathologic complete response in comparison to a pathologic complete response of 26% among patients who received surgery within 4–7 weeks (p = 0.02). The best available data based on these two studies suggest that surgery for patients who have completed neoadjuvant chemotherapy is recommended to proceed within 4–8 weeks.

In summary, delays in surgery ≥4 weeks for DCIS and invasive breast cancer, and ≥8 weeks for patients undergoing surgery following neoadjuvant chemotherapy, are associated with adverse outcomes.

#### iv Adjuvant and Neoadjuvant Therapy and Surgery Timing

In an effort to safely triage patients and conserve hospital resources and minimize the risk of COVID-19 among patients and staff, we considered chemotherapy alternatives and reviewed the literature for the impacts of chemotherapy on cancer patients with the infection. Unfortunately, the effect of chemotherapy induced immunosuppression on COVID-19 infection is mostly unknown to date. In a retrospective cohort study, including cancer patients with laboratory-confirmed COVID-19 infection from three designated hospitals in Wuhan, China, authors identified 28 cancer patients, 64% with Stage II and III solid tumors, with severe events during hospital admission [18]. Patients who received anti-tumor treatment within 14 days had a significantly increased risk of developing severe events (HR=4.079, P=0.037). Chavez-MacGregor *et al.* showed that time to chemotherapy >60 days influenced survival outcomes, particularly for patients with Stage III, triple-negative breast cancer, and HER2-positive tumors [19].

If surgery is delayed, we recommend three to six months of neoadjuvant endocrine treatment in all low risk ER-positive HER2-negative breast cancer patients [20, 21]. In HER2-positive breast cancer, less immunosuppressive regimens (e.g., paclitaxel + trastuzumab, trastuzumab emtansine) may be considered as an option over more suppressive regiments (e.g., docetaxel, carboplatin, trastuzumab and pertuzumab) [22–24]. The current data on this regimen show there may be more disease progression, but no difference in OS, and less toxicity, and therefore, it may be a therapy to consider for select patients [22–24]. Patients who are not candidates for neoadjuvant therapy should proceed with surgery if resources are available in their region. These are recommendations and are not intended to supersede individual physician judgement, nor institutional policy or guidelines.

### II Gastrointestinal Cancer

#### i Gastric Cancer

Gastric cancers have an estimated incidence of over 27,000 new cases in 2020, accounting for over 11,000 deaths [25]. Standard of care per the National Comprehensive Cancer Network (NCCN) includes multimodal therapy with neoadjuvant chemotherapy followed by surgery and adjuvant chemotherapy for T2 tumors and greater. T1a tumors can be treated with endoscopic resection, while T1b tumors have upfront radical resection or may benefit from perioperative chemotherapy followed by resection [26]. At the time of review, there are no studies that analyze delay in surgery after neoadjuvant chemotherapy for gastric cancer. There are studies evaluating the association of delay in surgery with the outcome in patients who have not undergone neoadjuvant chemotherapy, which are reviewed here.

Fujiya *et al.* evaluated the effect surgery wait time had on clinical Stage I gastric cancer in a retrospective review of 556 consecutive patients treated at the Shizuoka Cancer Center from 2007–2011 [27]. Delay was time from esophagogastroduodenoscopy to surgery and was stratified into three intervals: <61 days (short), 61–90 days (intermediate), and 91–180 days (long). The median age of the long wait time group was significantly older (p<0.001), and they had significantly more comorbidities (p=0.003); however, the staging and resection level (R0 vs. R1) was not significantly different between the groups. Median follow-up was 60.9 months with OS of 90.2%, 93.6%, and 88.8% in the short, intermediate, and long delay groups respectively. The HRs were not significantly different (p=0.22), and in multivariate analysis, age was the only variable that was significantly associated with OS. This study suggests that wait times up to 180 days with clinical Stage I gastric cancer does not increase mortality.

Furukawa *et al.* evaluated the impact of preoperative delay on OS for 696 patients with clinical Stage II and III gastric cancer who did not receive neoadjuvant chemotherapy from 2002–2012 [28]. The authors divided patients into three groups based on wait times: ≤30 days, <30 and ≥60 days, and >60 and ≤90. The groups demonstrated significantly different baseline characteristics, including lower body mass index (BMI), albumin, and hemoglobin in the short wait group (≤30 days), and more type 4 disease and higher pathologic Stage in the short wait group. On multivariate analysis, factors that were independently associated with survival included age >70 years, BMI >22 kg/m^2^, comorbidities, preoperative albumin of 4.0 g/dL, undifferentiated histology, preoperative macroscopic type 4 tumor, and advanced Stage but not preoperative wait time. This study shows that preoperative wait times up to 90 days do not result in worse OS for clinical Stage II and III gastric cancer.

These studies support that gastrectomy can safely be delayed for 2 to 3 months in patients with Stage I, II and III gastric cancer, but all gastric cancer patients should be evaluated for neoadjuvant therapy. Symptomatic patients with obstruction, perforation, and bleeding should not be delayed. We must consider that gastrectomy is a relatively morbid operation with reported complication rates ranging from 13–46% [29]. This could further make these patients immunocompromised as well as put them in an environment with greater viral transmission.

#### ii Hepatobiliary Cancer

Oncologic resections for hepato-pancreato-biliary cancers, similar to gastrectomy, are complex surgeries with a relatively high likelihood of morbidity and the potential for a prolonged length of stay and intensive care unit needs, which would place patients at high risk of COVID-19 infection and likely severe infection. We reviewed Hepatocellular Carcinoma and Pancreatic Cancer studies for associations of outcomes with increased time to surgery.

##### A Hepatocellular Carcinoma

Hepatocellular carcinoma (HCC) can be treated with ablation therapies in early Stage disease and otherwise are treated with surgical resection. Although surgery is the preferred treatment modality, the NCCN guidelines indicate that ablation for HCC ≤3 cm is an acceptable curative treatment regimen and could be considered for these patients to decrease delays. The impact of delays in surgical care for HCC and locoregional treatments such as arterial-directed and radiotherapies, used for patients with unresectable disease or as a bridge to a curative therapy, are reviewed here.

Mokdad *et al.* evaluated the impact of treatment delay on HCC through a retrospective review of a safety net hospital that implemented a notification system to the patients’ physicians via a voice messaging system [30]. Ninety-six patients included in the study were diagnosed with HCC 2 years prior to the intervention (45 patients) or 2 years after the intervention (51 patients). Characteristics between the two groups were similar, but the median time from diagnosis to treatment was significantly shorter after the voice messaging system intervention at 2.2 months vs. 5.5 months. Median OS was also significantly improved in the voice messaging system group at 28.5 months vs. 15.7 months. Time to surgery was not analyzed in a multivariable model, but these results at least suggest delays of greater than three months may worsen OS.

A retrospective cohort study by Singal *et al.* analyzed 267 patients with HCC to determine the factors associated with treatment delay and the impact of the delay [31]. HCC treatments were categorized as liver transplantation, resection, radiofrequency ablation, trans-arterial chemoembolization, systemic chemotherapy, or best supportive care. In patients who received multiple treatments, the time of treatment was the date of the first delivered treatment. Patients were dichotomized by a treatment delay of more or less than 3 months. When Barcelona Clinic Liver Cancer Classification and Child-Pugh class was adjusted for in the analysis, treatment delay was associated with significantly worse OS. Those without treatment delay had a 1- and 2-year survival of 89.8% and 64.5% respectively while those with a delay had 1- and 2-year survival of 63.7% and 50.1%. These results lead to the conclusion that a treatment delay of >3 months is associated with worse OS.

These studies suggest that patients with HCC should receive surgical treatment within 3 months to prevent cancer progression and decreased survival. If delay seems inevitable, neoadjuvant liver-directed therapy or systemic therapy may be a bridge to resection.

##### B Pancreatic Cancer

Treatment for pancreatic ductal adenocarcinoma includes either a surgery first approach or neoadjuvant chemotherapy. For resectable patients that are candidates for surgery first, or those that have completed neoadjuvant chemotherapy for pancreatic cancer, there are several studies that provide guidance on delay thresholds for surgery.

A multicenter randomized trial by Eshuis et al. evaluated the effects of surgical delay for pancreatic head cancer by comparing 185 patients randomized to surgery within 1 week versus preoperative biliary drainage with surgery at 4–6 weeks [32]. Median OS was not significantly different between the two groups nor was resection status (p=0.91 and 0.21 respectively). Multivariable Cox proportional hazard modeling, adjusting for patient and tumor factors revealed mortality was slightly lower with increased TTS. Results from this study suggest that a delay of 4–6 weeks with a biliary stent does not worsen OS.

Mirkin *et al.* used the NCDB to evaluate the association of OS with time to surgery for 14,807 patients with Stage I and II pancreatic ductal adenocarcinoma [33]. Patients were divided into quartiles for analysis: surgery within 1–2 weeks of diagnosis, 2–4 weeks, 4–8 weeks, and 8–12 weeks. On multivariable survival analysis, greater TTS was not associated with reduced OS. This model adjusted for patient, disease, and treatment characteristics, which incorporates bias due to adjustment for parameters in the putative causal chain. Taking these biases into account, this study suggests that patients treated within 12 weeks do not have worse survival.

Marchegiani *et al.* analyzed the effect delayed surgery influenced tumor size, pathological predictors of prognosis, and OS in 217 patients with resectable pancreatic ductal adenocarcinoma [34]. Patients were divided into two groups based on the time from diagnosis to surgery of ≤30 days and >30 days. Surgery delayed >30 days was associated with increased tumor size but no difference in R1 vs. R2 resection, vascular resection rates, T & N staging, perineural and lymphovascular invasion or local recurrence. Univariate analysis of OS showed that wait times of >30 days, >45 days, and >60 days were not significantly associated with OS. However, for tumor size less than 20 mm at diagnosis, early surgery was associated with decreased rates of nodal spread and significantly improved OS (p=0.02). This study provides some evidence that a delay >30 days for T1 tumors may worsen prognosis, while more advanced disease may have no difference in resectability, recurrence, or OS for surgery delays up to 60 days for resectable PDAC.

Rapitis *et al.* studied delay to surgery for 355 pancreatic ductal adenocarcinoma patients in the United Kingdom by measuring both delays to diagnosis and treatment. In this patient cohort, only 29% ultimately received an operation, and only 9% were resectable [35]. There was no significant difference in operability, resectability, and OS for patients that were diagnosed before or after their time point of 62 days from referral.

In summary, for resectable patients with pancreatic cancer, the data suggests that long term survival does not seem to be impacted by the delay in surgical resection, with the possible exception of patients with T1 tumors.

#### iii Colorectal Cancer

Colorectal cancer is the second leading cause of cancer-related death in the United States, estimated at 53,200, and over 145,000 new cases in 2020 [36]. The current standard of care for colon cancer is surgery followed by adjuvant chemotherapy for Stage III and considered for Stage II disease, while surgery alone is employed for earlier Stages [37]. For rectal cancer, the standard of care is neoadjuvant chemoradiation followed by surgery for Stage II or greater and surgery only for Stage I [38]. Some early Stage rectal cancer is amenable to endoscopic resection. This may be of consideration during the COVID-19 pandemic as it is an outpatient procedure that would have limited COVID-19 exposure to the patient if done at an outpatient surgical center. For colon cancer and rectal cancer patients who are candidates for surgical resection, there have been several studies evaluating the impact of delay in surgical treatment.

Hangaard *et al.* performed a systematic review in 2018 evaluating the impact of surgical treatment delays on survival in colorectal cancer patients [39]. Five observational studies were included for the analysis published between 2006–2017 with a total of 13,514 patients with delays ranging from 1 to ≥56 days. The time set as a delay was heterogeneous throughout the studies: two looked at quartiles, another divided delays of >60 days by type (total, provider, hospital), and another looked at >42 days and the 90^th^ percentile for the delay. None of the studies showed a significant association between treatment delay and survival. Based on this systematic review, surgical delays >60 days may not significantly worsen the prognosis for patients with colorectal cancer.

Bagaria *et al.* conducted a multi-institutional retrospective review of colorectal cancer patients at three hospitals in the Mayo Clinic network to study the association of OS and TTS for Stage II and III colon cancer from 1990–2012 [40]. The authors analyzed 4,685 consecutively treated patients and divided the patients into wait times by weekly increments from 1 week to >12 weeks. Patients with longer wait times were more likely older, male, single, and had a lower Charlson Comorbidity Index score. They also were more likely to have tumors with a lower grade and Stage. When delay to surgery was studied as a continuous variable, there was evidence of a trend towards worse 5-year survival with longer delays. Multivariable analysis showed increased mortality with a HR of 1.47 (95% CI 1.02–2.11, p=0.038) for delays >84 days but not an increase in upstaging with delay. The study concludes that mortality does not increase with delays up to 3 months, and that the increased risk of death after 84 days is unlikely to be due to progression of disease as the Stage was not shown to worsen with delay.

A retrospective analysis of the NCDB by Grass and colleagues from 2004–2013 analyzed the impact of delays from diagnosis to surgery in 118,504 patients with Stage I-III colon cancer who had ≤R1 resection [41]. Multivariable Cox regression model was used to study the influence delay had on survival along with patient and tumor factors. Delay was divided into lower and upper quartiles from diagnosis to treatment and was equivalent to <16 days and ≥37 days respectively. Patients in the shorter delay group had statistically significant more advanced disease (T, N, perineural invasion, and lymphovascular invasion all p<0.001), and the longer delay group had significantly more comorbidities (p<0.001). With a median follow-up time of 5.3 years, OS at 5 years was 75.4% and 71.9% in the short and long delay groups respectively, and 56.6% versus 49.7% at 10 years, which were both statistically significant at p<0.0001. Multivariate analysis showed that delay as a continuous variable was an independent predictor of OS with an HR of 1.06 (95% CI 1.05–1.07) for each 14-day delay, and OS began to decrease with delays longer than 30 days and reached significance at 40 days. This study suggests that delays >40 days worsen OS for patients with resectable Stage I-III colon cancer.

All these studies demonstrate that short delays, <40 days, do not have a significant impact on outcome, while longer delays, up to 3 months, have some evidence to suggest no significant change in outcomes. However, patients who are symptomatic with signs of obstruction, bleeding, or perforation should be operated on emergently.

### III Melanoma

Cutaneous melanoma is the fifth most common cancer worldwide for men and the sixth most common among women [42]. As treatment and cure of melanoma are largely based on resection, here we review studies evaluating time to surgery and the impact on melanoma outcomes.

A recent multivariate analysis of 153,219 melanoma patients queried from the NCDB revealed that significant differences in OS were not evident until time to treatment initiation was delayed for 90 or more days [43]. These results reaffirm the results of prior studies with smaller cohorts [44–46]. One notable finding in a study by Conic *et al.* was that Stage I melanoma patients did see worse OS with each additional month in definitive treatment delay from biopsy; the authors hypothesize that this is likely due to lower baseline mortality in Stage I disease, and any benefits in shorter time to treatment in Stage II or III disease were overshadowed by more dismal baseline prognosis [43].

Analyses of delay in time to sentinel lymph node biopsy (SLNB) are limited. One study compared 2483 primary cutaneous melanoma patients who either received early (<30 days) or delayed (>30 days) SLNB: there were no differences in melanoma specific OS or DFS, which reaffirms the results of the Multicenter Selective Lymphadenectomy 1 (MSLT 1) trial [47].

Current recommendations from the NCCN for the management of melanoma patients in the setting of the COVID-19 pandemic are in line with the available literature [48]. In brief, the NCCN recommends outpatient biopsy as soon as possible but delay of definitive wide local excision (WLE) for up to 3 months for melanomas ≤ 1mm thickness. T1 melanomas (<1 mm thickness), however, with positive margins, should be excised with appropriate margins in an outpatient setting. SLNB, which should be considered for patients with melanoma depth ≥0.8mm, may also be delayed for up to 3 months. As resource availability permits, T3/4 melanomas (>2 mm thickness) should take priority over thinner lesions, unless an incompletely biopsied T1/T2 melanoma has a large residual lesion. Consistent with MSLT-2 results, completion lymphadenectomy should be deferred despite positive SLNB results but with adequate clinical and radiographic follow-up as resources allow [48].

### IV Sarcoma

Soft tissue sarcomas are malignant mesenchymal tumors that comprise a heterogenous and aggressive subtype of cancer. They account for approximately 1% of adult solid tumors, with an estimated 12,750 new diagnoses in 2019. Featherall *et al.* reviewed the NDCB and had 648 patients meeting criteria with localized, high-grade soft tissue sarcoma diagnosed between 2004 and 2012 [49]. Treatment initiation included surgery, chemotherapy, and radiation, of which 87% had a surgery first approach. The 1-year, 5-year, and 10-year survival probabilities were obtained by specifying different times to treatment initiation values (TTI=14, 30, 60, 90, and 150), which showed no difference in OS.

Conversely, in osteosarcoma, a study from Beirut reviewed 38 pediatric patients <18 years old from 2001–2012 with localized disease [50]. Patients were given neoadjuvant chemotherapy with the goal of getting patients to definitive local control with surgical resection at week 10. Twenty-two out of the 33 patients that underwent surgery experienced delays by more than 4 weeks. The study reports surgery delayed >4 weeks corresponded to worse DFS. OS and DFS for patients with <4 weeks of delay to surgery was 80% for both (95% CI 45–95%). OS for patients with a delay of 4 weeks or more was 78% (95% CI 53–90%) while event-free survival was 58% (95% CI 36–76%, p<0.05).

In summary, patients with soft tissue sarcoma with longer time to treatment initiation intervals do not have direct negative prognostic implications, while pediatric patients with resectable osteosarcoma experienced worse survival when surgery is delayed by more than 4 weeks.

### V Well-Differentiated Thyroid Cancer

The incidence of thyroid cancer has been gradually increasing; however, fortunately, a majority of these cancers are not considered aggressive [51]. While there have been few studies specifically looking at delays in time of endocrine cancer surgery, guidelines have been developed to inform decisions on delaying surgery in pregnant patients with thyroid pathology [52–54]. These guidelines were based on studies that did not observe a worse prognosis or higher risk of recurrence from delaying thyroid surgery until postpartum [55, 56]. As a result, the American Thyroid Association states that surgical intervention for well-differentiated thyroid cancer is not required in pregnancy and that it can be monitored sonographically; but surgery should be considered if the tumor appears to grow more than 50% in volume or 20% in diameter by 24 weeks of gestation or if cervical lymph node cytology is malignant. Surgery in the second trimester is recommended however, if the differentiated thyroid cancer is advanced or if medullary thyroid carcinoma or anaplastic thyroid carcinoma is found on cytology or biopsy, delays in treatment will likely negatively impact outcomes [54]. These guidelines demonstrate that in well-differentiated thyroid cancer, surgery can be safely delayed for several months.

Additionally, delays in surgery in well-differentiated thyroid cancer outside of pregnancy have been studied and found to not significantly impact progression-free survival, recurrence, or DFS [52, 53]. The study by Amit *et al* [53] compared two patient subsets. Group one had benign cytology on initial biopsy with surgery delayed greater than 12 months at which time cancer was diagnosed. Group two contained patients who had cancer diagnosed on initial biopsy and underwent immediate surgery. The study found that delays did not negatively affect outcomes (p=0.09). Further, studies have investigated the utility of active surveillance as first-line management in select types of low-risk papillary thyroid cancer [57, 58]. Ito *et al.* defined low-risk papillary carcinoma microcarcinoma (< 1cm), while Tuttle *et al.* followed patients with tumors less than 1.5cm with minimal growth and no features concerning for invasion on imaging [57, 58]. Both studies reported active surveillance as a safe first-line management strategy for this disease due to its slow growth and stable progression over several years [57, 58].

The above studies demonstrate that well-differentiated papillary thyroid cancers were not significantly impacted by delays in surgery. It is, therefore, likely that, in the setting of the COVID-19 pandemic, surgical delays for most well-differentiated thyroid cancers will not significantly impact patient outcomes. It is important to clarify that these long-term studies recommending surveillance of “low-risk thyroid cancers” selected patients with tumors away from the trachea, thyroid capsule and isthmus and with clinically negative nodes. Therefore, size alone should not be used to select patients for delayed surgical intervention [59]. Of note, other more aggressive endocrine malignancies, such as medullary thyroid carcinoma, anaplastic thyroid carcinoma, and adrenocortical carcinoma, have little data on outcomes with regard to delays in surgery. Due to the frequency in which patients with these patients present with advanced stage or rapid progression, it is apparent that these are more urgent, and delays with these malignancies would likely be devastating [60–62].

### VI Genitourinary Cancer

#### i Prostate Cancer

Prostate cancer is the most commonly diagnosed cancer in men and the second leading cause of cancer death in men [63]. Low and intermediate-grade prostate cancers are typically indolent. Treatment options include radical prostatectomy, radiation therapy, androgen therapy, or active surveillance. It can be challenging to identify the true risk of surgical delay in prostate cancer, given the fact that trials of treatment vs. observation require over 10 years of follow-up to discern treatment benefit [64].

One large multi-institutional study of 813 low-risk and 748 intermediate-risk patients found that there was no negative impact of active surveillance in low-risk patients and an increased risk of biochemical recurrence for men with intermediate-risk cancers with delays >9 months [65]. Another study of 1,111 men with low-risk prostate cancers found a delay to surgery of >6 months was associated with higher rates of pathology upgrading and biochemical progression [66]. For men with high-grade cancers, one series which included 312 high-grade patients, found no difference in survival between those undergoing surgery within one month of diagnosis compared to those >70 days [67]. Another series which included 108 high-risk, 371 intermediate-risk, and 431 low-risk patients found no difference in biochemical recurrence whether surgery was performed within 6 months of diagnosis, at 6–12 months, or over 12 months [68].

These studies present sufficient evidence that a limited delay of radical prostatectomy for localized disease is oncologically safe. While there may be certain specific high-risk patients that would benefit from an earlier surgical intervention in an ambulatory surgical center, this must be carefully weighed against the opportunity cost of treating other patients with other more acute surgical diseases.

#### ii Renal Cancer

Renal cancers account for over 73,000 new cases per year in the US and nearly 15,000 deaths [69]. Fortunately, delaying radical nephrectomy for Stage II or higher renal cell carcinoma for up to 3 months had no survival impact in one series, and in another series, even delays of greater than 3 months did not impact cancer-specific survival [70, 71]. Thus, select renal cell carcinoma surgeries can be safely delayed in both low and high-COVID areas.

#### iii Bladder Cancer

Worse survival outcomes have been found for delays in treatment of Stage II or above urothelial carcinoma of the bladder. Specifically, surgery delays of over 3 months were shown to have worse OS in a recent SEER Medicare study of 1509 patients [72]. However, a meta-analysis showed that patients treated with neoadjuvant chemotherapy which were down staged had improved survival outcomes compared to radical cystectomy alone [73]. Therefore, in high COVID-19 areas, strong consideration should be given to neoadjuvant chemotherapy in candidates with histology responsive to systemic therapy and in those who can tolerate effective regimens. In low COVID-19 areas, a prompt radical cystectomy may be reasonable in appropriately selected patients.

### VII Ovarian Cancer

Ovarian cancer is the third most common gynecologic malignancy in the United States. In 2019, there were 22,530 estimated new cases and 13,980 estimated deaths from the disease [74]. Since the majority of women present with advanced-stage disease and undergo aggressive initial surgical cytoreduction, there is limited data measuring the impact of surgical delays on ovarian cancer survival. Most of the literature evaluates the impact of delay from the time of surgery to receipt of adjuvant chemotherapy, with even less data evaluating how a delay in primary cytoreductive surgery affects the outcome. This retrospective data is reviewed here.

A cohort study was conducted by Starbuck KD *et al.* from May 2006 to December 2016 involving 505 primary Stage IIIC/IV ovarian cancer patients who received platinum/taxane adjuvant chemotherapy following upfront cytoreductive surgery [75]. They compared on-time completion of adjuvant chemotherapy (105 days) to early finishing (<105 days) to delays of 1–4 weeks, and delays of >4 weeks. There was a statistically significant decrease in treatment response in those with delayed adjuvant treatment. Patients with delays in treatment had a complete response rate of 54.6% compared to those with no delays (83%) (p<0.001). Patients experiencing long delays of >4 weeks had significantly shorter median survival (18.1 months) compared to 43.1 months in the on-time adjuvant therapy group (p<0.001). Progression-free survival was also decreased with long treatment delays: 13.8 months vs 22.2 months in the on-time treatment group (p<0.001).

A single-center, retrospective cohort analysis performed at Shengjing Hospital of China Medical University between 2011 and 2015 included 489 patients with epithelial ovarian cancer who underwent upfront surgery followed by adjuvant therapy with taxane/platinum chemotherapy [76]. Time to chemotherapy greater than 28 days was associated with worse progression-free survival (HR: 1.36, 95% CI: 0.96–1.92) and OS (HR 1.38, 95% CI: 0.95–2.00). Advanced-stage patients with delayed time to chemotherapy had even worse progression-free survival (HR: 1.51, 95%CI: 1.02–2.24) and OS (HR: 1.53, 95% CI: 1.01–2.32).

A single-center, retrospective review by Liu Y *et al.* included 224 women with stage IIIC/IV ovarian carcinoma and assessed delays in both neoadjuvant chemotherapy and interval debulking surgery [77]. A total of 159 women underwent interval debulking surgery, and 34 experienced delays. While initial analysis associated delays with worse outcomes, after controlling for age, Stage, and complete gross resection, no significant impact was observed.

Surgery is utilized for diagnostic confirmation, staging, and initial treatment of ovarian cancer. In advanced ovarian cancer, it has been consistently demonstrated that optimal surgical cytoreduction is crucial to improving outcomes [78–80]. While some small reports have demonstrated that treatment delays do not significantly impact survival and have even correlated with improved survival, these are largely due to medical comorbidities or significant age differences of the patient population [81]. Patients with significant comorbidities or advanced age often need a longer time to recover from neoadjuvant therapy or for surgical optimization. In the case of a national health crisis, ovarian cancer patients who are currently healthy and/or optimized do not benefit from such delays and will ultimately decline in status with worsening symptoms or disease progression. Overall, ovarian cancer studies suggest that delays in surgery of greater than 4–6 weeks could negatively impact survival.

## Discussion

This multidisciplinary oncologic review of time to surgery shows that delays in oncologic surgery will likely negatively impact many tumor types. While there are some mixed results on the impact of delays on outcomes in each cancer subtype, cancers treated primarily with surgical management and those that are earlier Stage, may stand the most to lose from delays in treatment.

Some studies showed no significant difference or even improved survival with delayed surgery, but this could be a reflection of patients with significant comorbidities or advanced age that often need a longer time to recover from neoadjuvant therapy or for surgical optimization. In the case of a national health crisis, patients that are currently healthy and optimized do not benefit from such delays. In fact, many of these currently healthy, newly diagnosed patients will decline in status over a prolonged time period. This was observed in T1 pancreatic tumors and Stage I melanoma, where delays in treatment did not impact advanced disease outcomes but did impact early-stage disease outcomes.

As the COVID-19 pandemic reaches record infection rates and the resulting diminished healthcare resources mimic conditions in countries hit by economic crises and/or high healthcare burden, delaying oncologic surgery is likely to be considered or to occur [82, 83]. Low resources have been found to have significant impacts on emergent surgeries, including longer delays and increased mortality [82]. While oncologic surgeries are not typically considered emergent surgeries, evidence has shown that delays in cancer diagnosis and treatment in countries with low healthcare resources have significantly impacted patient outcomes [83, 84]. Surgical delays on a global scale could be devastating as reported in one study on 3,672,561 cancer patients that demonstrated that time to treatment increased the risk of mortality from 1.2–3.2% per week of delay in early stage cancers [85].

For patients that experience delay in surgery for their cancer due to COVID-19, neoadjuvant chemotherapy may be a treatment option for them to consider. However, chemotherapy also has risks of contributing to patient immunosuppression, predisposing them to more severe COVID-19 infection [86]. This would need to be an individualized discussion of the risks and benefits of pursuing neoadjuvant chemotherapy with a multidisciplinary cancer care team.

Limitations of this review on treatment delay include the potential for selection bias in retrospective studies. Retrospective studies are susceptible to the waiting time paradox, where more aggressive disease may be selected for earlier treatment. They are also subject to length time bias where patients with more aggressive diseases die or are no longer resectable as the time to surgery increases, and patients get selected out naturally. However, prospective randomized trials evaluating delay in surgery have not been performed as it would be unethical to randomize patients to a surgical delay. Therefore, the retrospective studies reviewed in this article represent the best available data on this topic. This study is also limited by interacting factors of surgical delay which were not uniformly adjusted for in the analyses of all these studies; these are variable that may be associated with surgical delay and cause worse outcomes independently, such as comorbidities, travel distance, socioeconomic status, and health literacy.

## Conclusion

This multidisciplinary oncologic review of time to surgery shows that delays in oncologic surgery will likely negatively impact outcomes for patients with multiple tumor types, with little to no impact on others. Our review includes breast cancer, gastrointestinal cancers (gastric, pancreas, hepatocellular cancer, and colorectal cancer), melanoma, sarcomas, well-differentiated thyroid cancer, genitourinary cancers (prostate cancer, renal cell carcinoma and bladder cancer), and ovarian cancer. This review demonstrates that time to surgery of more than 4 weeks negatively impacts survival in breast cancer, DCIS, pediatric osteosarcoma, T1 pancreatic cancer, Stage I melanoma, and patients with ovarian cancer coming off neoadjuvant chemotherapy. Decreased survival was also associated with surgical delays of three months or greater in patients with hepatocellular cancer, and 40 days in colorectal cancer. Studies do not demonstrate a significant impact on survival in gastric cancer, pancreatic cancer (>T1 tumors), advanced melanoma, soft tissue sarcoma, renal, prostate, and bladder, and well-differentiated thyroid cancer.

As there is still much unknown about the trajectory of the COVID-19 pandemic in the United States and worldwide, any specific triage system for non-emergent surgeries must be made with experienced oncology teams. This summary of the available data can aid in deciding which cancer patients should proceed to surgery amidst this pandemic. These decisions must be frequently re-evaluated as the circumstances surrounding a patient’s disease and the health system’s capabilities evolve.

## Abbreviations

DFS: Disease Free Survival
TTS: Time to Surgery
OS: Overall Survival
BCS: Breast Conserving Surgery
SEER: The Surveillance, Epidemiology, and End Results
NCDB: National Cancer Data Base
DCIS: Ductal carcinoma in situ
NCCN: National Comprehensive Cancer Network
HCC: Hepatocellular Carcinoma
SLNB: Sentinel Lymph Node Biopsy
